# Implantation of a new active bone conduction hearing device with optimized geometry

**DOI:** 10.1007/s00106-020-00877-2

**Published:** 2020-07-28

**Authors:** S. K. Plontke, G. Götze, C. Wenzel, T. Rahne, R. Mlynski

**Affiliations:** 1 Dept. of Otorhinolaryngology, Head and Neck Surgery, Martin Luther University Halle-Wittenberg, Ernst-Grube-Str. 40, 06120 Halle (Saale), Germany; 2 Dept. of Otorhinolaryngology, Head and Neck Surgery “Otto Koerner”, Rostock University Medical Center, Rostock, Germany

**Keywords:** Patients, Hearing aids, Otologic surgical procedures, Hearing loss, conductive, Hearing loss, mixed, conductive-sensorineural

## Abstract

Here, we describe the surgical technique for implanting a new, active, transcutaneous bone conduction hearing aid. The implant technology is based on a system that has been in use reliably since 2012. The geometry of the new implant has been adapted based on experience with previously introduced implants. The surgery was feasible, standardized, and safe. Due to the optimized geometric design that improved the bone fit, it is not necessary to use specialized, detailed preoperative planning, except in challenging anatomical conditions; e.g., in young children, malformations, poor pneumatization, or after a canal wall down mastoidectomy.

## Bone conduction hearing implants

Bone conduction hearing implants improve hearing in patients with conductive or mixed hearing loss, who do not experience hearing improvement with conventional hearing aids or conventional middle ear surgery (tympanoplasty, creation of an ear canal in atresia) [4, 8]. However, it is necessary to consider the limitations of these systems with respect to their audiological indication criteria [19]. In patients with single-sided deafness, bone conduction hearing implants can be used to rehabilitate hearing through “contralateral routing of signal” (CROS) [4, 23].

In the indications described above, bone conduction hearing implants have successfully restored hearing for decades with percutaneous mechanical energy transfer. However, the disadvantage of these implants is that they penetrate the skin, which poses the inherent risks of skin reactions and infections. Fussey et al. analyzed long-term effects of percutaneous bone conduction hearing implants in children. They reported that 77% of children experienced soft tissue complications that required treatment [10]. When soft tissue infections occur, the sound processor cannot be worn due to interference from the local treatment. This situation places constraints on hearing rehabilitation for the patient. A previous meta-analysis showed that these complications led to implant loss in 1.6–17.4% of patients [11].

With the introduction of minimally invasive implantation techniques and the avoidance of skin thinning with longer abutments, the complication rate appears to have improved. However, long-term follow-up studies in larger cohorts are lacking [24]. The typical soft tissue complications associated with percutaneous bone conduction hearing implants can be avoided with transcutaneous systems. With these systems, the active (vibrating) component is implanted subcutaneously, which avoids a percutaneous anchor. Additionally, the risk of skin irritation is reduced with inductive energy transfer, which avoids permanent pressure. One of these systems is the transcutaneous bone conduction implant (BCI) called “Bonebridge” (MED-EL, Innsbruck, Austria). The first generation (BCI 601) was introduced in 2012. In 2014, the BCI 601 was also approved for use in children, starting from age 5 years [3, 35].

The audiological outcome showed results similar to those reported for percutaneous bone conduction hearing implants [18]. However, the complication rate and the rate of revision surgery were lower with the transcutaneous system than with percutaneous implants [11, 18, 26]. These features have given rise to a trend towards using bone conduction hearing aid implants with transcutaneous inductive energy transfer, rather than percutaneous hearing aid implants [31].

The active (vibrating) component of the BCI 601 is responsible for energy transfer to the skull and, thus, to the inner ear. This component is called the bone conduction-floating mass transducer (BC-FMT). Based on three-dimensional (3D) reconstructions of temporal bones and “virtual surgery,” the BC-FMT can be adequately fitted to the mastoid bone in 77% of women and 81% of men, but less than 50% of children under 8 years of age [20]. Therefore, it was necessary to develop smaller active transcutaneous bone conduction hearing implants.

**Fig. 1:**
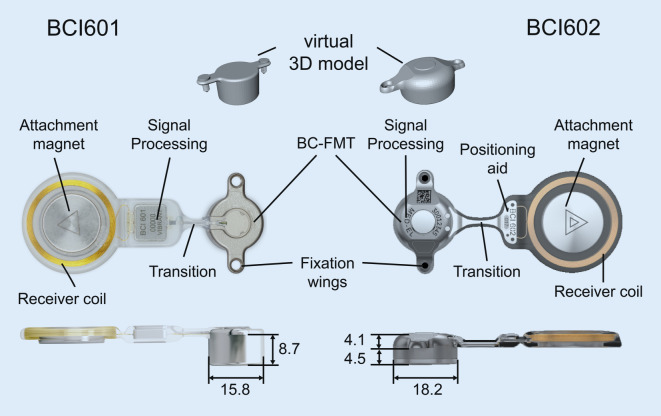
Comparison of the bone conduction implants (BCI) 601 and 602. Images show the new active bone conduction hearing system, BCI 602 (*right*), and the previous model, BCI 601 (*left*), (© MED-EL, Innsbruck, Austria, with permission). Virtual three-dimensional (*3D*) models of the bone conduction-floating mass transducers (*BC-FMT*) are used for preoperative planning. All measurements are expressed in millimeters

Further development of the BCI 601 (the BCI 602) reduced the penetration depth of the BC-FMT from 8.7 to 4.5 mm. This was achieved by increasing the BC-FMT diameter from 15.8 to 18.2 mm and locating some components of the BC-FMT above the bony surface. With the additional use of 1‑mm spacing washers (BCI 602 Lifts), the implantation depth can be further reduced to 3.5 mm. This configuration elevates the BC-FMT to 5.1 mm above the bony surface. The anchor holes in the fixation wings are 24.4 mm apart (BCI 601: 23.8 mm; Fig. 1).

This article describes the surgical technique for implanting the new active transcutaneous bone conduction hearing implant, BCI 602 (MED-EL, Innsbruck, Austria) with optimized geometry.

## Surgical technique

The implantable component of the bone conduction implant BCI 602 consists of an FMT with the electronics and an attachment magnet surrounded by the receiver coil (Fig. 1). The external part, the audio processor, is held in place by the magnet.

Preoperatively, the temporal bone is evaluated to determine placement of the BC-FMT. Several methods and tools have been described for this task, varying from a detailed visual analysis and two-dimensional (2D) measurements of standard computed tomography (CT) scans to sophisticated planning tools and preoperative “virtual surgery” [2, 6, 7, 12, 13, 15, 17, 27–30, 34].

**Fig. 2:**
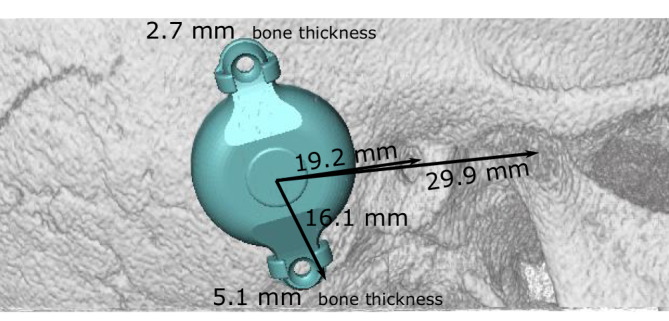
Landmarks for transferring the preoperatively determined implant position into the surgical field. Suitable landmarks include the mastoid tip, the anterior wall of the external ear canal or its remnants, the lateral orbital rim (not shown here), or the temporomandibular joint [17]. During planning, bone thickness is measured in the area where the self-drilling cortical screws will be placed to fix the bone conduction-floating mass transducer [33]

Surgery can be performed with either local or general anesthesia. All surgeries described here were performed with the patient under general anesthesia and additional periauricular infiltration with Ultracain/Suprarenin (articaine/adrenaline). The optimal position of the BC-FMT was then transferred to the surgical field (Fig. 2).

Surgery can be performed with either local or general anesthesia

**Fig. 3:**
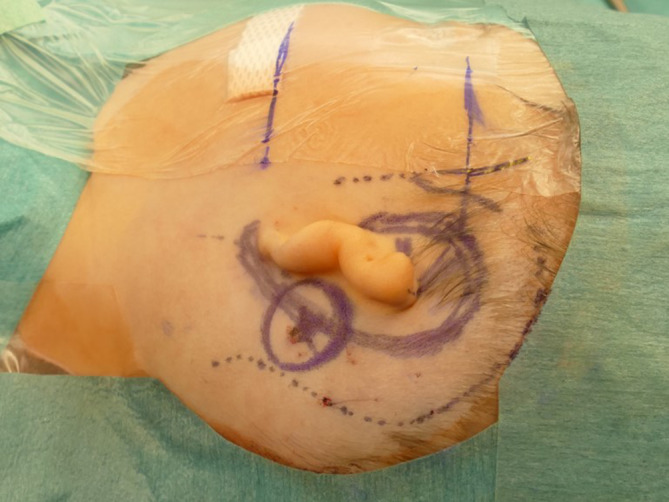
Planning the incisions. The skin and pericranial incisions should not be superimposed. Exceptions include cases of atresia, with a possible aesthetic auricular reconstruction planned at a later stage. In these cases, the position of the ear is marked according to standard recommendations. The posterior incision is made through all layers at a distance of approximately 15 mm from the later helix [9]

The surgeon should consider a number of precautions. First, the receiver coil and the audio processor should not come into contact with scars from previous surgeries. Pressure due to contact with the magnets could lead to impaired capillary perfusion and, in the worst case scenario, necrosis in the overlying scarred skin. Skin and pericranial incisions should not be superimposed. In cases where an aesthetic auricular reconstruction is planned at a later stage due to atresia, or to leave room for this possibility, a “posterior atresia incision” can be performed, as described by Frenzel et al. (2010). In this procedure, an incision cuts through all layers, following which a subperiosteal dissection is performed. Thus, the physical integrity of the tissue layers around the ear remnant are preserved (Fig. 3; [9]).

**Fig. 4:**
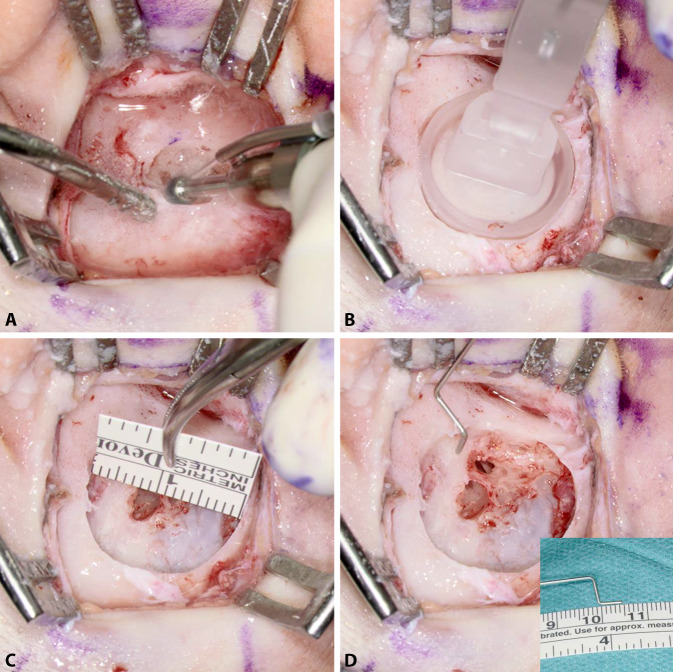
Creating the implant bed. **A** Drilling of the implant bed is started in the center of the position planned for the bone conduction-floating mass transducer. The depth is 4.5 mm and the diameter is relatively small, approximately 5 mm. The implant bed is successively enlarged by moving the drill bit away from critical structures (sigmoid sinus, dura, ear canal, open mastoid cavity) to avoid damage. **B** The adequacy of the size and shape of the implant bed can be evaluated with a sterile “BCI 602 Sizer Kit.” Alternatively, the implant bed can be measured with a sterile ruler and a depth gauge (here, the authors used a 0.7-mm suction tip, off-label) (**C,** **D**)

Next, only the implant bed for the BC-MFT is drilled. Despite careful preoperative planning, the implant bed might reach the dura, the sigmoid sinus, an ear canal remnant, or an open mastoid cavity. Therefore, the authors typically start by drilling the implant bed in the center of the BC-FMT position to a depth of 4.5 mm with a small-diameter drill bit, approximately 5 mm in diameter. The implant bed is then gradually enlarged. By successively moving the drill bit away from the structures mentioned, they can be avoided (Fig. 4**A**). The adequate size and shape of the implant bed can be evaluated with a sterile “BCI 602 Sizer Kit” (Fig. 4**B**). Alternatively, an adapted sterile ruler and an intraoperatively “created” depth gauge (the authors use a 0.7-mm suction tip, off-label) have proven useful for this measurement (Fig. 4**C**, **D**).

**Fig. 5:**
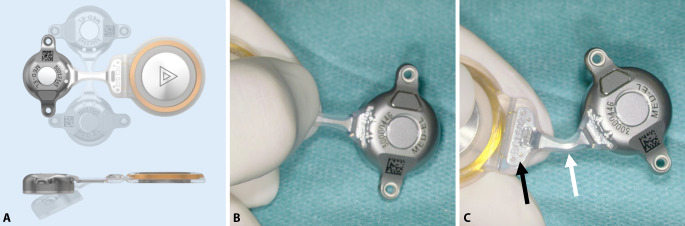
Positioning the receiver coil and magnet. **A** The implant can be bent at the transition zone (*white arrow* in **C**) ±90° in the horizontal plane and −30° in the vertical plane. While bending, the implant should be held with the thumbs and index fingers of both hands; one hand should be placed at the positioning aid (**B** and *black arrow* in **C**), and the other hand should be placed at the bone conduction-floating mass transducer. (**A**: MED-EL, Innsbruck, Austria, with permission)

**Fig. 6:**
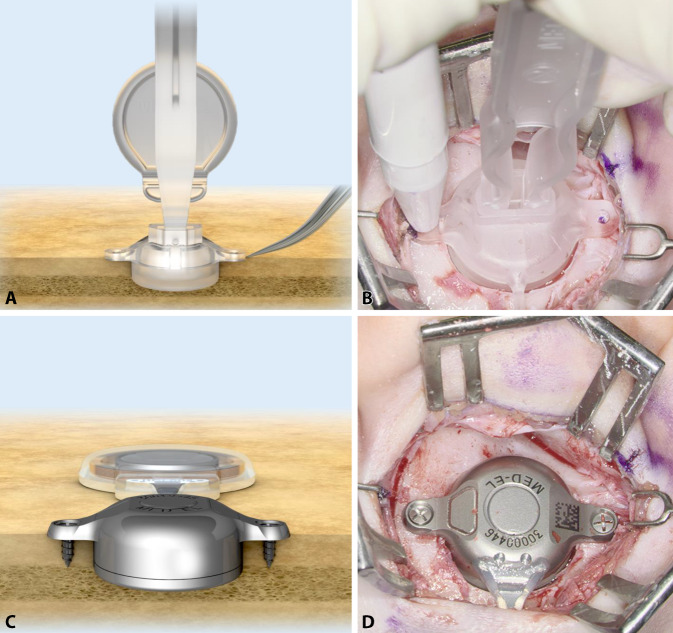
Fixing the bone conduction-floating mass transducer (BC-FMT) to the bone. **A,** **B** The fixation wings with anchor holes should lay flat on the bone. When pressing the BC-FMT down with a finger, it should not wiggle or wobble. **C,** **D** The BC-FMT is fixed with self-drilling cortical screws; this procedure is simpler in this model (BCI 602) than in the previous model (BCI 601). (**A**, **C**: MED-EL, Innsbruck, Austria, with permission)

The implant can be bent in the transition zone, ±90° in the horizontal plane and −30° in the vertical plane (Fig. 5). While bending, the implant should be held with the thumbs and index fingers of both hands, one at the positioning aid and one at the BC-FMT (Figs. 1 and 5). The receiver coil and the attachment magnet are placed in a subperiosteal pocket, directly on the skull. The fixation wings with anchor holes should lay flat on the bone (Fig. 6**A**, **B**). When pressing the BC-FMT down with a finger, it should not wiggle or wobble. The BC-FMT is then fixed with self-drilling cortical screws (Fig. 6**C**, **D**).

The audio processor retention force is determined by the thickness of the skin over the magnet and receiver coil and by the magnet strength [32]. For the BCI 602, skin thickness should not exceed 7 mm; this can be tested with a skin flap gauge [1]. Skin thickness can also be determined by putting an injection needle through the skin in the area where the receiver coil and magnet will be placed.

By successively moving the drill bit away from the structures mentioned, they can be avoided

Skin closure is typically performed in layers, with resorbable (subcutaneous and pericranial: 3/0) and non-resorbable (skin, 4/0) sutures. Alternatively, cyanoacrylate topical skin adhesive can be used to close the skin. The pericranial layer can usually be closed completely over the BC-FMT, despite its partial elevation above the bone surface.

The first activation and fitting of the audio processor is carried out after complete disappearance of any swelling over the receiver coil and magnet, at approximately 4 weeks after surgery, in an appropriate audiological center. Thereafter, an adaption phase of several weeks is necessary, depending on the individual patient. Technical and medical check-ups are necessary at least once per year.

**Fig. 7:**
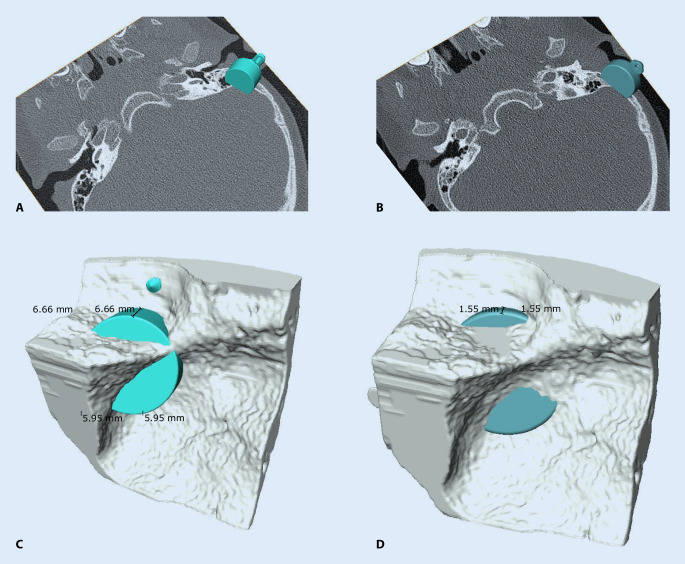
Preoperative plan for the implantation of an active, transcutaneous bone conduction hearing system in a 4.5-year-old patient with atresia and complete conductive loss (according to Plontke et al. 2014 [17]). The bone conduction-floating mass transducers required intracranial penetrations of: (**A,** **B**) 6–7 mm for the BCI 601 and (**C,** **D**) <2 mm for the BCI 602. Due to the expected skull growth [25], a computed tomography scan and planning were not repeated before surgery at the age of 5.5 years. Intraoperatively, the dura and sigmoid sinus were exposed, but not impressed. This patient is also shown in Fig. 4

Figure 7 shows the preoperative planning for implanting bone conduction hearing systems in a 4.5-year-old patient with atresia (ear canal stenosis, malformation of the malleus and incus, and a thickened stapes footplate) and complete conductive hearing loss. The parents declined the offer to continue hearing rehabilitation with a soft-band bone conduction hearing aid or canaloplasty with tympanoplasty. Preoperative 3D planning was performed without BC Lifts, since lifts would further elevate the BC-FMT over the bony surface, a feature the authors like to avoid, particularly in the presence of thin skin, which is common in children.

The audio processor is first fitted and activated at an audiology center following wound healing

**Fig. 8:**
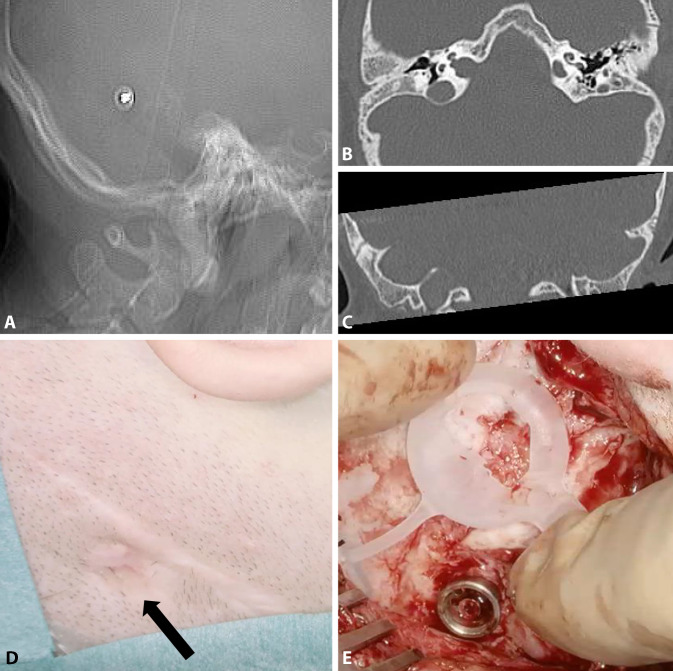
Radiological planning and surgical field for the simultaneous explantation of a fixture from a percutaneous bone conduction implant and implantation of a BCI 602 (right ear). **A** The computed tomography (CT) scout view shows the fixture. Axial CT (**B**) and coronal (**C**) CT show mastoid hypoplasia, the lateralized sigmoid sinus, and minimal cortical bone thickness (<5 mm) at the bone conduction-floating mass transducer (BC-FMT) position. The abutment is completely overgrown with skin (*black arrow*) (**D**) and is removed before creating the implant bed (**E**). (**A–C**: CT images reproduced with permission from Prof. Dr. med. M.A. Weber, Institute for Diagnostic and Interventional Radiology, Pediatric and Neuro-Radiology, University Medicine Rostock)

**Fig. 9:**
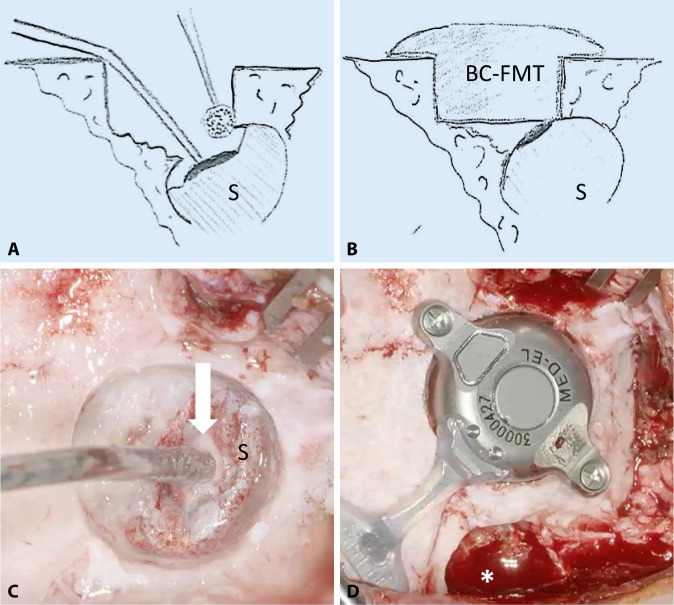
Placing the bone conduction-floating mass transducer (*BC-FMT*) close to the sigmoid sinus. **A,** **C** Due to the close proximity of the sigmoid sinus (*S*), a bony island is created on the exposed sinus wall (*white arrow*). This can be used to protect the sinus from the drill bit by temporarily and gently pressing on the island with the suction tip. **B,** **D** Final position of the BC-FMT after removing the pre-existing fixture (*asterisk*)

Figure 8 shows the preoperative planning for an adult patient with bilateral complex malformation and after previous hearing rehabilitation with a percutaneous bone conduction hearing implant on the right side (BAHA, Cochlear, Sydney, Australia) and a transcutaneous system on the left side (BCI 601). The patient had experienced recurrent skin irritation around the abutment, including skin overgrowth; therefore, the percutaneous bone conduction system on the right side was replaced with a BCI 602. Detailed preoperative CT-based planning was required due to scars from the previous skin incisions, the plan to simultaneously explant the fixture, and the presence of mastoid hypoplasia. Placing the BC-FMT required limited exposure and temporary impression of the sigmoid sinus (Fig. 9). By pressing the bony island of the sinus inward with a suction tip, it could be protected while the BC-FMT implant bed was molded with the burr. This technique can also be applied to avoid injury to the dura.

## Discussion

The dimensions of the BC-FMT are based on physical and technical requirements. The previous model (BCI 601) was associated with considerable risk of depressing the sinus and/or the dura, particularly in children, malformations, poorly pneumatized mastoids, and after canal wall down surgery.

To avoid depressing the sinus or dura, CT-based surgical planning is recommended

Therefore, detailed preoperative, CT-based surgical planning was recommended in those situations [2, 6, 7, 12, 13, 15, 17, 20, 22, 27–30, 34]. The introduction of BCI Lifts increased the probability that the BC-FMT could be fitted to the bone with 4‑mm lifts in up to 100% of patients older than 9 years [20, 28].

A recent meta-analysis that focused on the BCI 601 showed a rate of 1.7% (five of 289 implantations) for serious adverse events (SAEs), i.e., complications that led to surgical revision or device explantation [14]. A previous retrospective, monocentric observational study reported that the BCI 601 was explanted in five of 64 patients (7.8%) due to implantation out of audiological (one patient) or anatomical indication criteria (four patients). Three patients experienced protrusions into an open mastoid (“radical”) cavity, and one patient received an implantation under the scar tissue from a previous BAHA implant [5]. Another retrospective, multicenter observational study reported SAEs in three of 61 (5%) patients. One patient required inpatient treatment due to a procedure-related wound infection and a history of multiple previous reconstruction surgeries for dysplasia and atresia. The second patient complained of cephalalgia, which, during revision surgery, was found to be due to newly formed soft tissue and bone between the implant and the dura. In the third patient, the device was exchanged due to an unanticipated device-related SAE (device failure) at 27 months after the primary surgery [18].

The lower penetration depth of the BC-FMT into the temporal bone requires a larger BC-FMT diameter

At the authors’ centers, only one of 51 (2%) patients required a BCI 601explantation. However, that was not device-related, but due to sudden hearing loss in the contralateral ear and, thus, the patient lost the audiological indication range for a CROS. Two revision surgeries (3.9%) were necessary; one was to reduce the skin flap over the receiver coil and magnet, and the other was to relocate the implant bed due to progressive outlining of the implant through the preexisting thin retroauricular skin, without skin irritation. Two other patients experienced infections of the auricular episthesis anchors; however, the infections were not related to the bone conduction hearing implant.

The BCI 601 and 602 manufacturer has specified that the audiological indication limit is a maximum bone conduction threshold of 45 dB, in the frequency range of 0.5–to 3 kHz. Considering the maximum power output and a dynamic range of 30–35 dB, and based on the authors’ own experience, the best audiological results (sufficient loudness and dynamic range) are achieved when bone conduction has not yet reached the 45-dB limit [16, 19, 21]. For single-sided deafness, the bone conduction threshold should not exceed 20 dB, in the range of 0.5–3 kHz, in the contralateral (hearing) ear (based on manufacturer specifications and the authors’ own experience).

**Fig. 10:**
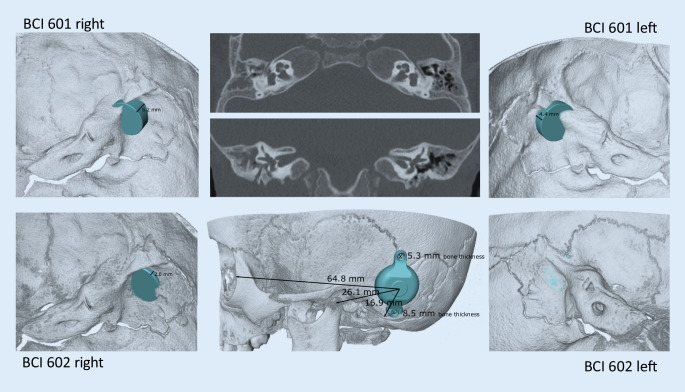
Preoperative planning based on Plontke et al. 2014 [17] for the implantation of the active transcutaneous bone conduction hearing systems, BCI 601 and BCI 602, in a 3-year-old patient. The patient had bilateral atresia, poorly pneumatized mastoids, hypoplastic tympanic cavities, dysplastic ossicles, an atypical facial nerve course, and complete conductive loss. Without the BCI Lifts, the BCI 601 required intracranial penetrations of 6.2 mm (*BCI* *601 right*) and 4.4 mm (*BCI* *601 left*). The new implant with optimized geometry (BCI 602) required intracranial penetration, with an impression of the dura and/or sinus, of 2 mm (*BCI* *602right*). However, intracranial penetration was completely avoided on the left side by using a 1-mm BCI Lift at the inferior fixation wing (*bottom row center*). After informed consent about the off-label use in this age group, and due to the explicit wishes of the parents, the BCI 602 was implanted in this patient at the age of 3 years and 4 months (this patient is also shown in Fig. 3). The axial computed tomography (CT) (*center top*) and coronal CT (*center middle*) are reproduced with permission from Prof. Dr. S. Kösling (Radiology, University Medicine Halle, Germany)

With the new active BCI and its optimized geometry, the anatomical indication range has significantly increased compared to the previous model (BCI 601). A study that performed “virtual surgery” in 151 temporal bones of 81 children and young adults (ages 5 months–20 years) demonstrated that, in all patients aged 12 years and older, the BCI 602 could be completely fitted to the bone. In patients aged 3–5 years, the BCI 602 could be fitted to the bone in 75% of cases. In contrast, a complete bone fit was not achieved with the BCI 601 in any of the temporal bones without BCI lifts [33]. However, the BCI 602 has not been approved for this age group of up to 5 years. A lower penetration depth would entail a reduced volume of the BC-FMT. However, adequate acceleration of the skull, which is necessary for stimulating the inner ear, would be difficult to achieve. The resonance frequency is indirectly, exponentially related to the mass of the BC-FMT (~m^−1/2^). Thus, for an electromagnetic transducer with a given mass and resonance frequency, a lower penetration depth required a larger diameter (BCI 601: 15.8 mm and BCI 602: 18.2 mm; Fig. 1). This corresponds to a 15% increase in the diameter and a 45% reduction in the volume of the implant bed. The latter was reached by partially translocating the BC-FMT above the skull surface (Fig. 1). Due to the translocation of the electronics (demodulator) into the BC-FMT, the overall “footprint” of the implant has decreased. Consequently, depending on the individual configuration of the temporal bone, implantation in children under 5 years old appears to be possible and has been performed (off-label) in individual cases (Fig. 10).

## Summary

- The new active bone conduction hearing implant technology is based on a system that has been reliable since 2012.
- The surgery is feasible, standardized, and safe.
- The optimized geometric design has improved the fit of the implant to the bone even under challenging anatomical conditions.
